# Part-Aware Mask-Guided Attention for Thorax Disease Classification

**DOI:** 10.3390/e23060653

**Published:** 2021-05-23

**Authors:** Ruihua Zhang, Fan Yang, Yan Luo, Jianyi Liu, Jinbin Li, Cong Wang

**Affiliations:** 1 School of Computer Science (National Pilot Software Engineering School), Beijing University of Posts and Telecommunications, Beijing 100876, China; rhzhang@bupt.edu.cn (R.Z.); luoyan88228@bupt.edu.cn (Y.L.); wangc@bupt.edu.cn (C.W.); 2 Key Laboratory of Trustworthy Distributed Computing and Service, Beijing University of Posts and Telecommunications, Beijing 100876, China; 3 School of Electronics Engineering and Computer Science, Peking University, Beijing 100871, China; fyang.eecs@pku.edu.cn; 4 School of Cyberspace Security, Beijing University of Posts and Telecommunications, Beijing 100876, China; 5 Local Servive Center, National Population Health Data Center, Beijing 100005, China; lijinbin301@126.com

**Keywords:** thorax disease classification, soft attention, mask-guided attention, multi-task learning

## Abstract

Thorax disease classification is a challenging task due to complex pathologies and subtle texture changes, etc. It has been extensively studied for years largely because of its wide application in computer-aided diagnosis. Most existing methods directly learn global feature representations from whole Chest X-ray (CXR) images, without considering in depth the richer visual cues lying around informative local regions. Thus, these methods often produce sub-optimal thorax disease classification performance because they ignore the very informative pathological changes around organs. In this paper, we propose a novel Part-Aware Mask-Guided Attention Network (PMGAN) that learns complementary global and local feature representations from all-organ region and multiple single-organ regions simultaneously for thorax disease classification. Specifically, multiple innovative soft attention modules are designed to progressively guide feature learning toward the global informative regions of whole CXR image. A mask-guided attention module is designed to further search for informative regions and visual cues within the all-organ or single-organ images, where attention is elegantly regularized by automatically generated organ masks and without introducing computation during the inference stage. In addition, a multi-task learning strategy is designed, which effectively maximizes the learning of complementary local and global representations. The proposed PMGAN has been evaluated on the ChestX-ray14 dataset and the experimental results demonstrate its superior thorax disease classification performance against the state-of-the-art methods.

## 1. Introduction

Chest X-rays (CXR) have been one of the most common radiological examinations aiding in thorax disease diagnosis [1,2]. While leveraging CXR images, most existing diagnostic methods still rely on the radiologist, who need to observe carefully to read the image. However, the lack of professional radiologists limit people’s access to thorax disease screening, especially during the pandemic, such as the SARS in 2003 and the COVID-19 pandemic of 2019. On the other hand, CXR images contain complex pathologies and subtle texture changes of different thorax diseases, which bring great challenges to disease diagnosis even for professional radiologists, thus may lead to wrong diagnosis. Aiming to address these challenges, it is important to develop the CXR image classification systems to support the daily clinical routines.

**Figure 1:**
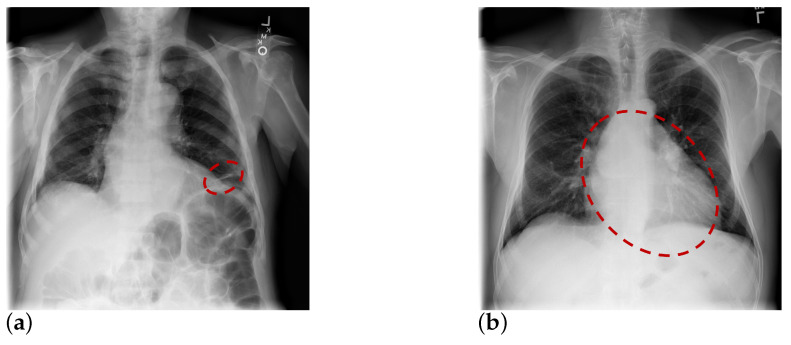
Motivations and concepts behind the proposed part-aware mask-guided attention in thorax disease classification: Pathological changes usually only happen in the local regions of CXR image, the corresponding thorax diseases thus only relate to specific organs. (**a**) A CXR image has ‘Atelectasis’, (**b**) A CXR image has ‘Cardiomegaly’.

Deep neural networks have been widely used for medical image analysis [3,4,5,6,7] as well as thorax disease classification tasks in recent years. Leveraging large-scale CXR datasets such as ChestX-ray8 [8] and ChestX-ray14 [9], they detect pneumonia from CXR by automatically learning the feature representations of X-ray images based on a supervised learning paradigm. Most existing deep learning-based approaches directly learn a global feature representation from a whole CXR image for thorax disease classification, but without considering in depth the informative local regions. For example, several works adopt prevalent Convolutional Neural Network (CNN) models, i.e., ResNet [10] and DenseNet [11], to classify multiple thoracic pathologies according to information that is captured from global CXR image. Although these methods have achieved some promising results, exploiting informative regions to learn discriminative local features from CXR images remains an open research challenge. A complete CXR image mainly consists of different organs, such as the left-lung, right-lung and heart, where some organs may have pathological changes and have some specific diseases. For example, atelectasis is usually related to only lungs and cardiomegaly is usually related to only the heart as illustrated in Figure 1. To capture the local information, some approaches [12,13] design a multi-channel CNN architecture that learns complementary local features from some cropped local regions. On the other hand, the local region generation technique usually suffers from misalignment problem and introduces extra computation.

Visual attention is a mechanism that guides the feature learning toward informative regions by indicating where the important cues are lying for a certain task. Given a specific learning object, it helps the deep models to learn target relevant feature representations by generating an attention map where non-informative regions usually have much weaker response compared with regions of interest. Due to its good potential to train a better deep model, visual attention has been widely used for various computer vision tasks, i.e., image classification [14] and person re-identification [15], and has brought significant performance improvements. Visual attention also has been applied in thorax disease classification in recent years, but most existing methods [16,17,18,19,20] learn global attention maps from whole CXR images only where local informative cues lying around specific organs are often suppressed. To refine the learned global feature, several works [16,17,19] have been reported to either use single attention module or use multiple attention modules to re-weight the learned feature representations of backbone network. In addition, ref. [20] adopts a multi-branch attention network to capture richer global feature representations. To capture disease-specific local features, Huang et al. [18] learn a multi-attention network, in which each attention map presents the most informative regions related to each category. However, the produced attention maps are also global since they are learnt from the whole CXR image. In addition, since a deep network usually learns feature representation and attention map simultaneously supervised by a single task driven objective function, it might be biased to capture too many background cues when CXR images have very complex backgrounds. These cues dramatically introduce difficulties when extracting robust feature representations and thus compromise the thorax disease classification performance.

Motivated by the experience of expert radiologists who leverage both global (contextual) and local (saliency) cues simultaneously to analyze the CXR images, we aim to learn both global and local features from CXR images for optimal thorax disease classification. Specifically, global features from whole CXR images lay the groundwork, and local features from organ images capture complementary information. Robust local feature learning requires good localization of human organs, which can be located by human organ masks obtained from anatomical segmentation models [21]. However, to the best of our knowledge, only one work [13] has adopted organ masks into thorax disease classification. This is mainly due to the fact that anatomical segmentation models bring large computational complexity. Inspired by this observation, we propose an innovative Part-Aware Mask-Guided Attention Network (PMGAN), which learns complementary global and local feature representations guided by visual attention networks. Specifically, we introduce a segmentation constraint using an organ mask to regularize the learning of the attention module, in this way the organ mask is only needed in the training stage, which will not introduce extra computation during the inference stage.

The reset of this paper is organized as follows. Section 2 introduces some related works. Section 3 presents PMGAN in detail and our experimental results are showed in Section 4. At last, the summary of this work is presented in Section 5.

## 2. Related Work

Deep learning has been widely studied for years in medical image analysis and a number of technologies have been reported in the literature. This section will introduce related deep learning-based thorax disease classification methods since our approach is CNN based. According to different learning strategies, current deep learning-based methods can be broadly grouped into three categories including: (1) thorax disease classification using global information [9,22,23,24,25,26,27,28], (2) thorax disease classification using global and local information [12,13] and (3) thorax disease classification using visual attention [16,17,18,19,20].

### 2.1. Thorax Disease Classification Using Global Information

Since the 2012 ImageNet challenge [29], where AlexNet architecture [30] improved the accuracy remarkably for image classification, CNN has been proved to be a very powerful tool to deal with computer vision and pattern recognition problems, and becomes prevalent in visual feature representation learning. For the thorax disease classification task, earlier researchers directly adopt off-the-shelf CNN architectures, i.e., ResNet [10] and DenseNet [11], to learn global feature representations from whole CXR images. For example, Wang et al. [9] adopted AlexNet [30], GoogLeNet [31], VGG16 [32] and ResNet50 [10] as feature extraction networks, respectively. The feature extraction network is first pre-trained on ImageNet [29]. Then they remove the last fully connected layers and the final classification layer, targeting the pre-trained backbone. In addition, they add a transition layer, which is followed by a global pooling layer and a prediction layer to form the complete model for thorax disease classification. Wang et al. [25] propose a TieNet, which introduces the text embedding of radiological reports to improve the thorax disease classification accuracy. Rajpurkar et al. [23] design a 121-layer CheXNet which has been announced to outperform radiologists in their ability to detect 14 thoracic diseases on ChestX-ray14 [9] dataset. Aiming to capture detail information from original High-Resolution (HR) CXR images, Ranjan et al. [22] introduce an auto-encoder structure into the CNN model, which jointly learns thoracic disease classification and image reconstruction. Specifically, they use a learnable auto-encoder to reduce the resolution of original CXR images rather than simply use interpolation techniques, which may result in the loss of detail cues, thus severely hindering thorax disease classification. Pant et al. [33] adopt a residual UNet to replace generic CNN structure for pneumonia diagnoses. Chen et al. [24] combine two asymmetric subnetworks (ResNet [10] and DenseNet [11]) to adaptively capture discriminative feature representations of different abnormalities from the CXR images. Yao et al. [27] use LSTMs to leverage interdependencies among target labels under the assumption that multiple diseases classification contains rich relationship information among pathologies. Chen et al. [26] bring Graph Convolution Networks (GCNs) into thoracic disease classification to explore the correlation information of pathologies. More recently, Gündel et al. [28] incorporate the lung/heart segmentation task into the thorax disease classification system to regularize the feature representation learning.

Though these approaches can capture global CXR image feature representations effectively and improve the thorax disease classification accuracy significantly, they often ignore local cues which are essential for some diseases with only small pathological changes and thus lead to suboptimal classification performance.

### 2.2. Thorax Disease Classification Using Global and Local Information

With the goal of addressing the problem of methods learning global feature representation only, there are new methods proposed to jointly learn complementary global and local features from CXR images. Designing multi-channel CNN architecture is a common approach to learn multi-granularity feature representation. Multi-channel CNN architecture has been adopted for various tasks [34,35,36,37,38,39] in computer vision and pattern recognition research communities due to its good potential to learn complementary feature representations. For example, Wu et al. [34] propose an MM-CNN which learns feature representations of industrial process data along the time dimension by a multi-channel and multi-head CNN. They capture features of industrial process data from the local to the global level, for use in fault classification. Lyu et al. [35] adopt the multi-channel CNN architecture to improve the performance of ultrasound tomography image reconstruction. Cheng et al. [36] propose a multi-channel parts-based CNN to learn global features from whole-body images and local features from body-part images for person re-identification. Over the past few years, several multi-channel CNN based models [12,13] have been proposed for learning global and local feature representations for thorax disease classification. For example, Wang et al. [12] propose a two-branch CNN architecture including: (1) one global branch that learns features from global images and (2) one local branch that learns features from a local region, which is guided by heatmaps produced by class activation mapping (CAM). Liu et al. [13] propose a two-branch CNN model where one branch is used to capture the features of the whole CXR images and the other branch is used to obtain features of the cropped lung region images. Note that the local lung regions are generated by a segmentation network, which introduces extra computation.

Although a number of multi-channel CNN based methods have been proposed, exploiting accurate organ images to learn detail cues remains an open research challenge. In addition, most existing methods adopt off-the-shelf CNN architectures to design multi-channel network without considering the relationship among branches. More importantly, they miss in-depth examination of specific organ images and ignore the importance of different regions within both global images and organ images while learning feature representations.

### 2.3. Thorax Disease Classification Using Visual Attention

Recently, a number of methods use visual attention to optimize deep neural networks for thorax disease classification [16,17,18,19,20]. For example, Sorkhei et al. [19] add a space attention module on top of a pre-trained ResNet to capture global context features, which are then combined with original feature maps (local features). In addition, they introduce an attention gated module after the second and third residual blocks of ResNet to progressively refine the learning feature representation from coarse to fine. Ma et al. [20] propose a cross-attention model that first leans two feature maps by using two independent attention networks. Then, an element-wise hadamard product is adopted to fuse these two feature maps to produce cross-attention feature maps. Wang et al. [16] propose a triplet attention model that simultaneously learns the channel-wise, element-wise, and scale-wise attention to capture discriminative information for the thorax disease classification task. Huang et al. [18] propose multiple attention modules that learn multi-attention maps simultaneously. They optimize each attention module by corresponding disease label and thus each attention map consists of feature representations that are related to each category. Ma et al. [17] propose a multi-attention learning framework for comprehensive thoracic disease classification and localization, which consists of a feature attention module, a space attention module and a hard example attention module. Specifically, the feature attention module is a squeeze-and-excitation structure, which is equipped after each residual block of ResNet101 to refine the extracted feature maps at multiple resolutions. The space attention module consists of a global average pooling layer and a resize operation, which is used to enlarge the receptive field of final classifier and bring global information. A hard example attention module is proposed to alleviate the class imbalance problem by increasing the proportion of positive examples.

A visual attention mechanism has been proven to be an efficient technique in feature representation learning [40,41,42]. However, on the one hand, most existing approaches mainly focus on learning the attention map using global CXR images, without considering learning dedicated attention map from each local organ region. On the other hand, global attention tends to guide feature learning toward the global salient regions which often suppresses local informative regions around organs, and thus leads to suboptimal thorax disease classification performance when CXR images have very complex backgrounds.

Aiming to address above constraints, the proposed part-aware mask-guided attention network learns global and local feature representations from both global and local informative regions. First, it adopts multiple mask-guided attention for accurate organ detection. Second, it learns complementary attention maps from global CXR images and precisely located organ images. Moreover, two independent binary cross-entropy classification losses are introduced to optimize attentive global and local branches independently and concurrently, with the aim of maximize the learning of complementary local and global feature representations.

## 3. Methodology

The thorax disease classification task is defined as: Given N CXR images $I={\left\{I_{i}\right\}}_{i=0}^{N-1}$ in which each image is labeled with *q* thorax diseases $L_{i}=\left[l_{i}^{1},l_{i}^{2},...,l_{i}^{j}\right]$ (where $l_{i}^{j}\in \left\{0,1\right\},j=0,...,q$), the objective of thorax disease classification is to learn a model that has the capability of correctly classifying each CXR image into categories of corresponding *q* thorax diseases. Thus, thoracic disease classification is a multi-label classification problem.

**Figure 2:**
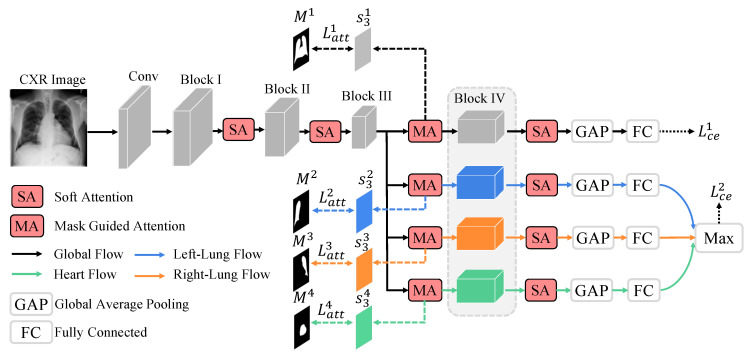
The framework of the proposed part-aware mask-guided attention network (PMGAN). Given a CXR image $I_{i}$, features are first sequentially extracted by the Conv1 layer and residual blocks I–III from low-level to high-level. Blocks I and II are followed by a dedicated Soft Attention (SA) module to refine the corresponding feature representations. An all-organ region and three single-organ regions are then determined by four independent Mask-Guided Attention (MA) module. Four dedicated attentive branches based on residual Block IV are further designed to map the respective input region to feature representations.

We propose a novel Part-Aware Mask-Guided Attention Network (PMGAN) that learns complementary global and local features from whole CXR images and local organ images independently and concurrently for thorax disease classification, as illustrated in Figure 2. The following subsections will present the design of PMGAN, the baseline model, the soft attention module, the part-aware mask-guided attention module and the loss functions in detail.

### 3.1. Baseline Model

We adopt the ResNet50 [10] as our base network which consists of a conv layer and four residual blocks (Blocks I–IV) as illustrated in Figure 2. For a given CXR image $I_{i}$, a conv layer is first employed to capture low-level features, then four consecutive residual blocks are adopted to further capture high-level semantic features progressively. On top of residual Block IV, a global average pooling layer is first applied to the learned global feature representation to obtain a feature vector $v_{i}$. A *q*-dimensional fully-connected layer is then applied to obtain the output $Y_{i}=\left[Y_{i}^{1},Y_{i}^{2},...,Y_{i}^{q}\right]$, which is the predicted probability of *q* thorax diseases. Finally, a sigmoid activation layer is employed to normalize the output $Y_{i}$ to range [0,1] as follows:

(1) $$Y_{i}^{j}=Sigmoid\left(w^{j}v_{i}+b^{j}\right),j=0,...,q,$$

where $w^{j}$ and $b^{j}$ denote the weight vector and bias terms of the prediction function for the *j*-th disease. Table 1 presents the detailed configuration of the base network.

We use the binary cross-entropy loss to train the base network. Given a training CXR image $I_{i}$ with *q* thorax disease labels $L_{i}^{j},j=0,...,q$ and $Y_{i}^{j}$ denoting the output of the network, the binary cross-entropy loss $L_{\mathrm{ce}}^{1}$ can be defined as follows:

(2) $$L_{\mathrm{ce}}^{1}=-\frac{1}{N}\sum_{i=1}^{N}\frac{1}{q}\sum_{j=1}^{q}\left[L_{i}^{j}log\left(Y_{i}^{j}\right)+\left(1-L_{i}^{j}\right)log\left(1-Y_{i}^{j}\right)\right],$$

where *N* is the number of training images, *q* is number of disease classes.

### 3.2. Soft Attention

Aiming to refine the extracted features across multiple resolutions, we design multiple soft attention modules that re-weight the learned feature representations of residual blocks I–IV progressively as illustrated in Figure 2. Specifically, Let $f_{i}\in R^{h\times w\times c}$ denote the feature maps extracted by the *i*-th residual block, where *h*, *w* and *c* denote the height, width and channel of $f_{i}$, respectively. Let $m_{i}\in R^{h\times w\times c}$ (with the same size as $f_{i}$) denotes the attention maps estimated by the soft attention module that follows the *i*-th residual block. With the feature maps $f_{i}$ and attention maps $m_{i}$, we adopt a residual attention scheme [14] to re-weight the feature maps $f_{i}$ as follows:

(3) $${\hat{f}}_{i}=\left(1+m_{i}\right)⊗f_{i}$$

where ${\hat{f}}_{i}$ denotes the adjusted feature maps, ⊗ denotes element-wise product. As defined in Equation (3), the features are largely enhanced when the attention scores of corresponding positions approximate 1. Otherwise, they remain almost unchanged when the corresponding attention scores approximate 0.

Note that the Mask-Guided Attention (MA), after residual block III, has the same structure as the soft attention, the only difference is that it is constrained by organ masks. More details of the proposed mask-guided attention module will be described in the next subsection.

**Figure 3:**
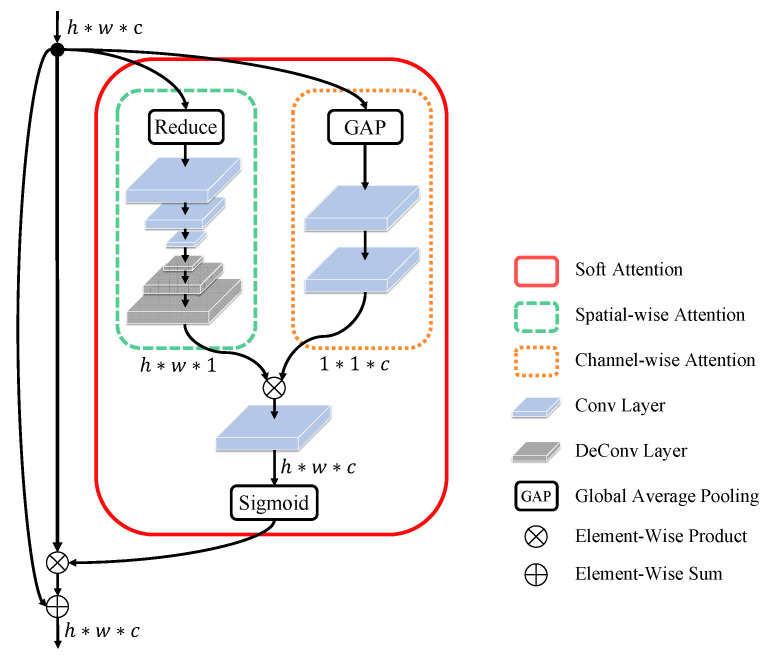
Architecture of the proposed soft attention network: Given the feature maps $f_{i}$ that extracted by the *i*-th residual block, a spatial-wise attention sub-network is adopted to estimate an attention map $s_{i}\in R^{h\times w\times 1}$, while a channel-wise attention sub-network is applied to estimate an attention map $t_{i}\in R^{1\times 1\times c}$. Spatial-wise and channel-wise attention maps are then fused by an element-wise production operation for feature re-weighting.

Aiming to reduce the number of parameters and lower the optimizing complexity, we split the soft attention network into two sub-networks, one for spatial-wise attention network and the other for channel-wise attention network as illustrated in Figure 3. These two attention sub-networks estimate the attention scores concurrently and independently. Specifically, the spatial-wise attention network estimates an attention map $s_{i}\in R^{h\times w\times 1}$ (with the same spatial size as $f_{i}$) of the *i*-th residual block, in which each attention confidence score indicates the importance of each spatial image region. All features therefore share the same spatial attention map. For example, the heart region is important when the CXR image has cardiomegaly. The channel-wise attention network estimates an attention map $t_{i}\in R^{1\times 1\times c}$ (with the same size of channel as $f_{i}$) of *i*-th residual block, in which each attention confidence score indicates the importance of each semantic feature. For example, the shape feature is the most important visual cue around the heart region while predicting cardiomegaly. The spatial-wise and channel-wise attention networks thus guide the learning to capture most important features from semantic-related regions simultaneously for optimal thorax disease classification. More details of the proposed soft-attention network will be presented in the following subsections, including spatial-wise attention network and channel-wise attention network.

#### 3.2.1. Spatial-Wise Attention

The spatial-wise attention network consists of a feature reduction layer and an encoder-decoder structure as illustrated in Figure 3. In particular, the feature reduction layer is a global average pooling operation that compresses the feature maps $f_{i}$ of *i*-th residual block across the channel dimension as follows:

(4) $$f_{i}^{\mathrm{spatial}}=\frac{1}{c}\sum_{j=1}^{c}f_{i,1:h,1:w,j}$$

As studied in [43], since all visual cues share the same spatial attention map, the feature reduction layer will not deteriorate the attention learning. Moreover, it reduces the parameters of the following layers by $\frac{1}{c}$ times since the input size h×w×c is compressed to h×w×1.

On the other end, the encoder–decoder structure attempts to extract multi-scale feature representations for comprehensive attention estimation. It is inspired by the human visual system that first perceives the whole image in a large reception field and then progressively focuses on the salient local regions for discriminative visual cues capturing. Specifically, the encoder consists of several conv layers (each with stride 2 and kernel size 3×3) that process the input feature map down to a predefined lowest resolution. Afterward, the decoder consists of several deconv layers (with the symmetrical structure as encoder) that iteratively generate a pixel-wise attention map (with the same spatial size as the input feature map). For the encoder, note that we apply 3 conv layers in Block I, 2 conv layers in Block II, and 1 conv layer in Block III–IV.

#### 3.2.2. Channel-Wise Attention

A feature channel can be interpreted as a semantic feature that captured by a conv filter across the spatial domain. The learning of the channel-wise attention can be interpreted as a process of selecting the most discriminative features with respect to the all spatial image regions. All spatial image regions therefore share the same channel attention map. We first apply a global average pooling layer to the input feature maps $f_{i}$ to obtain a channel feature $f_{i}^{\mathrm{channel}}$ as follows:

(5) $$f_{i}^{\mathrm{channel}}=\frac{1}{h\times w}\sum_{j=1}^{h}\sum_{k=1}^{w}f_{i,j,k,1:c}.$$

Two convolutional layers are then adopted to process the channel feature $f_{i}^{\mathrm{channel}}$ to obtain a channel-wise attention map $t_{i}\in R^{1\times 1\times c}$ as follows:

(6) $$t_{i}=ReLu\left(BN\left(W_{2}\left(ReLU\left(BN\left(W_{1}f_{i}^{\mathrm{channel}}\right)\right)\right)\right)\right),$$

where $W_{1}\in R^{\frac{c}{r}\times c}$ and $W_{2}\in R^{c\times \frac{c}{r}}$ denote the parameters of the first and second convolutional layers, *r* denotes the reduction factor that is used to reduce model complexity. In our implementation, *r* is empirically set at 16.

#### 3.2.3. Combination of Spatial-Wise and Channel-Wise Attention

The spatial-wise attention map $s_{i}$ and channel-wise attention map $t_{i}$ are combined by an multiplication operation followed by a convolutional layer (with kernel size 1×1) to produce the final attention map $m_{i}$ as follows:

(7) $$m_{i}=Conv\left(s_{i}\times t_{i}\right)$$

Finally, a sigmoid activation layer is employed to normalize the output $m_{i}$ to range [0,1].

### 3.3. Part-Aware Mask-Guided Attention

**Figure 4:**
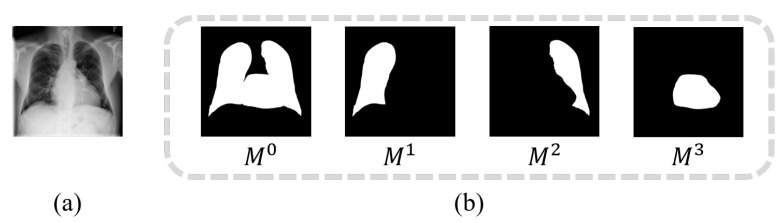
Illustration of the organ segmentation: (**a**) Input CXR image, (**b**) four generated organ masks.

As illustrated in Figure 2, aiming to extract richer visual cues from local regions for thorax disease classification, we first design four dedicated Mask-guided Attention (MA) modules $A_{b}\left(b=0,...,3\right)$ to process the input feature maps $f_{3}$ (output of residual Block III) to produce organ-related features. Four network branches with independent parameters then further learn higher-level global and local features from previous organ-related features. Based on the observation that organs can be precisely localized by organ masks, we adopt anatomical segmentation techniques [21,44] to first automatically generate organ masks and then use the generated organ masks to constrain the attention learning of the proposed MA. In particular, we employ an off-the-shelf segmentation method [21] to generate four organ masks, the all-organ mask $M^{0}$, the left-lung mask $M^{1}$, the right-lung mask $M^{2}$, and the heart mask $M^{3}$ as illustrated in Figure 4. Since the mask-guided attention network has the same architecture as soft attention network, we introduce independent segmentation constraint into each MA to guide the attention learning toward corresponding organ region. Specifically, with the organ mask $M^{b}\left(b=0,...,3\right)$ and spatial-wise attention map $s_{3}^{b}$ (generated by *b*-th MA after 3-rd residual block), the segmentation constraint is computed by Root Mean Squared Error (RMSE) as follows:

(8) $$L_{\mathrm{att}}^{b}=\sqrt{\frac{\sum_{j=1}^{h}\sum_{k=1}^{w}{∥M_{j,k}^{b}-s_{3,j,k}^{b}∥}^{2}}{N}},$$

where *N* is the number of training images.

The overall segmentation constraint can thus be defined as follows:

(9) $$L_{\mathrm{att}}=L_{\mathrm{att}}^{0}+\beta \sum_{b=1}^{3}L_{\mathrm{att}}^{b},$$

where β controls the relative weights of segmentation constraints of global and local branches.

An important point to note here is that anatomical segmentation model brings large computational complexity. Since we only use the segmentation constraint (as defined in Equation (8)) in the training stage, the anatomical segmentation model is used only during training to generate the organ masks. In the inference stage, our proposed approach therefore dose not add any computation that introduced by the anatomical segmentation model. Such comprehensive attention modeling elegantly guides the network branches to learn feature presentations from precisely localized organ regions in training stage and without introducing computation during the inference stage.

### 3.4. Loss Functions

As illustrated in Figure 2, four prediction results are obtained according to their corresponding features relate to all-organ region, left-lung region, right-lung region and heart region, respectively. Moreover, a max score operation is applied to the local branches that aim to select the most relative organ for each disease. Aiming to maximize the learning of complementary global and local features from all-organ and single-organ regions for optimal thorax disease classification, two independent binary cross-entropy loss are adopted to supervise the feature learning of global and local branches. Thus the binary cross-entropy loss $L_{\mathrm{ce}}^{2}$ of local branches can be defined in a similar way as Equation (2) as follows:

(10) $$L_{\mathrm{ce}}^{2}=-\frac{1}{N}\sum_{i=1}^{N}\frac{1}{q}\sum_{j=1}^{q}\left[L_{i}^{j}log\left(Z_{i}^{j}\right)+\left(1-L_{i}^{j}\right)log\left(1-Z_{i}^{j}\right)\right],$$

where *q* is number of disease classes, $Z_{i}^{j}$ is the probability of *j*-th thorax disease relates to *i*-th CXR image as predicted by local branches.

The overall classification loss can thus be derived by combining the global binary cross-entropy loss $L_{\mathrm{ce}}^{1}$ (as defined in Equation (2)) and local binary cross-entropy loss $L_{\mathrm{ce}}^{2}$ as follows:

(11) $$L_{\mathrm{ce}}=L_{\mathrm{ce}}^{1}+\alpha L_{\mathrm{ce}}^{2},$$

where α controls the relative weights of binary cross-entropy losses of global and local branches which is set to 0.5 in our implementation.

The objective function of the part-aware mask-guided attention network can be derived by combining the binary cross-entropy loss $L_{\mathrm{ce}}$ with the segmentation constraint $L_{\mathrm{att}}$ as follows:

(12) $$L=L_{\mathrm{ce}}+L_{\mathrm{att}}.$$

## 4. Experiments

### 4.1. Dataset and Settings

#### 4.1.1. Dataset

**Figure 5:**
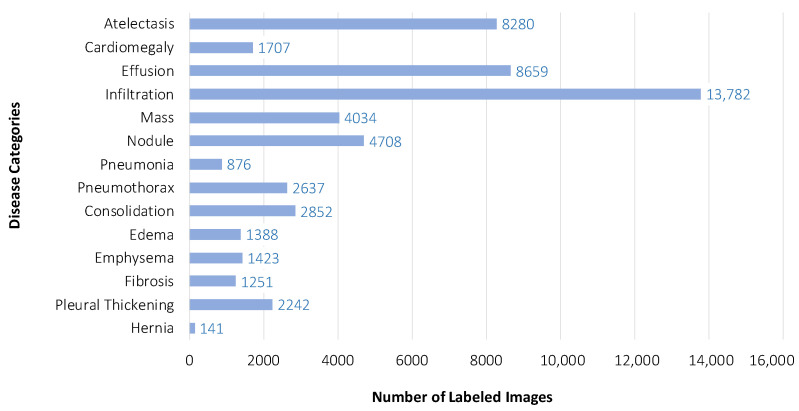
Distribution of 14 thorax disease labels on the ChestX-ray14 dataset.

We evaluated our proposed Part-Aware Mask-Guided Attention Network (PMGAN) on the ChestX-ray14 dataset, which is an extension of ChestX-ray8 dataset released in [8]. ChestX-ray14 is a commonly used benchmark dataset for thoracic disease classification task. It consists of 112,120 frontal-view Chest X-ray (CXR) images of 30,805 unique patients with 14 disease classes. These 14 disease classes are Atelectasis, Cardiomegaly, Effusion, Infiltration, Mass, Nodule, Pneumonia, Pneumothorax, Consolidation, Edema, Emphysema, Fibrosis, Pleural Thickening, and Hernia. The thoracic disease classification is thus a multi-label classification task. This dataset provides CXR images in PNG format with 1024×1024 resolution. The dataset also provides meta data including: 14 diseases labels, patient ID, patient age, patient gender and view position. For the 112,120 CXR images, 60,412 of them are labeled as ‘No Finding’ (without any diseases), while the others are labeled with up to 14 thorax diseases. Figure 5 presents the label distribution of 14 thorax diseases on the ChestX-ray14 dataset. In our all experiments, we followed the official protocol in [8] which randomly selects 70% of the images for training, 10% for validation and the rest 20% from testing. In addition, we also evaluated our proposed PMGAN using *k*-fold cross-validation method. In our implementation, *k* was empirically set at 5. More specifically, the ChestX-ray14 dataset was randomly partitioned into five equally-sized subsamples, where each subsample consisted of 22,424 CXR images. For the five subsamples, a single subsample was used as testing data, and the remaining four subsamples were used as training data. The cross-validation process was then repeated five times, with each of the five subsamples used exactly once as the validation data. The five results were then be averaged to produce the final evaluation performance.

#### 4.1.2. Evaluation Protocol

We employed the Area-Under-Curve (AUC) to evaluate the performance of the proposed PMGAN. AUC is a widely used metric for binary classification problems including the thorax disease classification performance problem. Specifically, *q* ROC (Receiver Operating Characteristic) curves for *q* disease classes are first plotted to measure the thorax disease classification performance at various threshold settings. The AUC is then used as a summary of each ROC curve to measure the ability of the classifier to distinguish between classes. The higher the AUC, the better the classifier is at distinguishing between CXR images with the thorax disease and no disease.

#### 4.1.3. Implementation Details

Our proposed PMGAN was implemented on the PyTorch framework. Specifically, the PMGAN was first initialized with the weights that pre-trained on ImageNet [29]. It was then fine-tuned on the ChestX-ray14 dataset by the Adam optimizer [45], where the batch size is set to 128, the weight decay was set to $1\times 10^{-4}$, the learning rate was set to 0.0001. We fine-tuned the network with a maximum number of epochs of 100 and early stop the training when the validation error improvement was below a threshold with a patience of 5 epochs. All CXR images were rescaled to 512×512, and each image was first normalized by subtracting its channel means and then dividing its channel standard deviations. In the inference stage, we first predicted the classification score by using both global and local branches simultaneously. A max score operation is then applied to the scores predicted by global and local branches to obtain the final score.

### 4.2. Comparison with State of the Arts

The proposed PMGAN was evaluated and compared with most state-of-the-art thorax disease classification methods on widely used dataset ChestX-ray14. Specifically, PMGAN was compared with 11 state-of-the-art methods including: (1) eight methods using global information only (Wang [9], AECNN [22], CheXNet [23], DualCheXNet [24], TieNet [25], CheXGCN [26], Yao [27] and Gündel [28]), (2) two methods using both global and local information (ThoraxNet [12] and SDFN [13]), and (3) five methods using visual attention ($A^{3}$Net [16], Ma [17], Huang [18], AG [19] and CAN [20]). Table 2 and Table 3 show the experimental results. As Table 2 and Table 3 show, PMGAN obtained superior thorax disease classification accuracy and outperformed state-of-the-art approaches by 1.5% in average AUC. The significant performance improvements demonstrate the importance of learning complementary global and local features from informative all-organ region and single-organ regions using comprehensive attention. In particular, PMGAN improved average AUC by 2.37% as compared to the CheXNet [23] which learns global feature only. In addition, PMGAN improved average AUC by 1.5% as compared to the method proposed by Huang et al. [18], which uses the global visual attention only. By taking a second look, it can be observed our proposed PMGAN achieves comparable performance when evaluated using *k*-fold cross-validation method as compared with using hold-out method. This demonstrates the robustness of our proposed model.

### 4.3. Ablation Study

Our proposed PMGAN learns complementary global and local feature representations for comprehensive thoracic disease classification. The soft attention is designed to guide feature learning toward informative regions. In addition, multiple part-aware mask-guided attention network branches are designed to learn feature representations from an all-organ region, left-lung region, right-lung region and heart region, respectively. To find out how each of these two innovative attention modules helps to improve the thorax disease classification performance in Table 2 and Table 3, We developed three models for ablation study including (1) a baseline model which implements the base ResNet50; (2) a soft attention (SA) model that includes the soft attention beyond the baseline; (3) a mask-guided attention (MA) model that includes the part-aware mask-guided attention network beyond the SA model.

Table 4 presents the results about the performance of the 3 models on the ChestX-ray14 dataset. As Table 4 shows, the inclusion of soft attention significantly helps to improve the thorax disease classification performance. Specifically, SA improves average AUC by 1.32% as compared to the baseline. Especially in Herina, SA outperforms baseline by a large margin (3.84% in AUC). This demonstrates the effectiveness of learning feature representations from informative regions for thorax disease classification problem. More specifically, MA consistently outperforms SA, which is largely due to the incorporation of the complementary local features.

**Figure 6:**
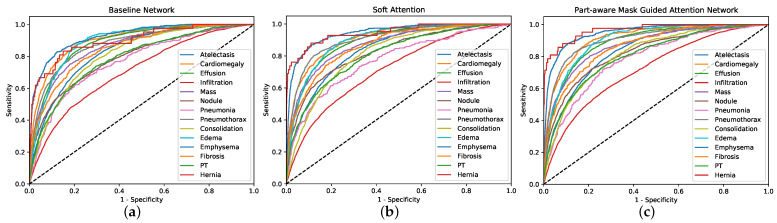
The ROC curves on the 14 diseases. (**a**) The ROC curves of baseline network, (**b**) The ROC curves of soft attention network, (**c**) The ROC curves of part-aware mask-guided attention network.

Figure 6 further illustrates how our proposed PMGAN improves the baseline network that does not include soft attention and part-aware mask-guided attention. In the figure, we plot the ROC curves of baseline, soft SA and MA on the 14 diseases ChestX-ray14 dataset.

### 4.4. Discussion

In addition to the ablation study, we also studied three factors that could affect the thorax disease classification performance including: (1) the inclusion of mask-guided attention after different residual blocks, (2) the use of multiple binary cross-entropy loss, (3) the hyper-parameters, and (4) the computation of segmentation constraint with different losses.

#### 4.4.1. Mask-Guided Attention Analysis

Our PMGAN consists of four branches including: (1) a global branch that learns feature representation from all-organ region, and (2) three local branches that learn feature representation from left-lung region, right-lung region and heart region, respectively. Since the lower convolutional layers extract low-level patterns that are common to all semantic structures in the same CXR image, the global and local branches share the shallow layers to reduce the number of model parameters and over-fitting risks. On the other hand, the model with more shared lower layers will limit the representation capability of higher layers, which may deteriorate the thorax disease classification performance. We evaluate how the sharing granularity of global and local branches affects the thorax disease classification performance, in cases where the mask-guided attention module is appended to different residual blocks.

As mentioned in Section 3.2, the Mask-Guided Attention (MA) has the same structure as the Soft Attention (SA), and the only difference is that it is constrained by organ masks. In our implementation, we adopt segmentation constraint (as defined in Equation (8)) to guide the attention learning of different SA modules that are appended to different residual blocks. A SA module with segmentation constraint is named to MA. Since the experiments here are to evaluate how the sharing granularity of global and local branches affects the thorax disease classification performance, the global and local branches only share the layers before MA module; and do not include MA and following layers. As Table 5 shows, the best performance is obtained when mask-guided attention module is appended to residual Block III.

#### 4.4.2. Multi-Task Learning

One key idea in the training of the proposed PMGAN is to optimize the global and local branches independently with multiple losses to maximize the learning of complementary global and local features from all-organ region and single-organ regions. We investigate how this multi-task learning approach helps to improve the thorax disease classification performance as compared with the traditional feature representation learning with single loss. As can be seen from Table 6, when the PMGAN is trained by the multi-task learning strategy with multiple binary cross-entropy losses, it achieves significant performance improvement.

#### 4.4.3. Parameter Analysis

**Figure 7:**
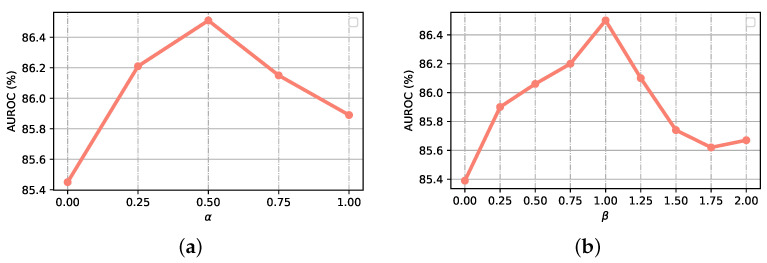
Evaluation with different values of α and β.

We first evaluate the impact of α (as defined in Equation (12)) which controls the relative weights of binary cross-entropy losses of global and local branches. As illustrated in Figure 7a, the inclusion of multiple binary cross-entropy losses clearly improves the thorax disease classification performance (as compared with the inclusion of single loss when α is set to 0). The best thorax disease classification performance is archived when α=0.5. We also evaluate the impact of β (as defined in Equation (9)) which controls the relative weights of segmentation constraints of global and local branches. Figure 7b shows the thorax disease classification performances with different values of β. As illustrated in Figure 7b, a moderate β helps to enhance classification capability of PMGAN, and the best performance is obtained when β is set to 1.0.

We also compare the proposed PMGAN with the baseline model (ResNet50 [10]) in CPU computational complexity. As Table 7 shows, our PMGAN only doubles the computational complexity, though it consists of four branches. The fair computational complexity is largely due to the four branches of PMGAN share the first conv layer and three residual blocks (Blocks I–III) as illustrated in Figure 2. Additionally, the soft attention and part-aware mask-guided attention are both computational light and do not introduce much computational overhead.

#### 4.4.4. Segmentation Constraint Analysis

The segmentation constraint can be computed by different losses, such as Binary Cross Entropy (BCE) loss, dice loss, as well as Root Mean Squared Error (RMSE). We investigate how different losses affect the thorax disease classification performance. As Table 8 shows, the thorax disease classification performance of PMGAN is impacted just marginally and the best results are obtained when the PMGAN is trained by RMSE. The experiment results demonstrate that different losses result in subtle numerical differences in segmentation constraint, and may not affect the attention learning significantly.

#### 4.4.5. Contribution and Difference from Previous Works

In this paper, we propose an Part-Aware Mask-Guided Attention Network (PMGAN), which explicitly enforces the complementary global and local feature learning in an attentive manner. More specifically, there are two stages in our network. In the first stage, an attentive CNN branch is proposed to learn the global feature and attention maps from whole CXR images. In the second stage, a multi-branch attentive network is designed to learn the global and local feature representations as well as attention maps simultaneously, in which each branch is guided by corresponding organ mask. The proposed PMGAN has four major contributions as listed:

- It designs a novel multi-branch network architecture that learns complementary global and local feature for thorax disease classification under the guidance of organ masks;
- It designs a novel mask-guided attention network that learns features from precisely located all-organ and single-organ regions concurrently and independently;
- It designs a novel multi-task independent learning scheme to maximize the learning of complementary local and global representations by optimizing multiple losses on the same disease label concurrently;
- It develops an end-to-end trainable deep network that achieves superior thorax disease classification performance.

Indeed, a number of deep learning based approaches [9,12,13,22,23,24,25,26,27,28] have been reported, but exploiting organs to capture local cues for optimal thorax disease classification remains an open research challenge. For our part, by observing that some diseases only occur at a specific organ and some diseases may occur at different organs, we design a part-aware multi-branch network that learns multi-granular feature representations from all-organ region and single-organ regions simultaneously for thorax disease classification.

While visual attention has been used in existing methods [16,17,18,19,20], we here explore the usability of visual attention in multi-granular feature representation learning. More specifically, for the all-organ region and each of the interested single-organ regions, a dedicated attentive network branch is designed to learn the optimal feature representations and attention maps simultaneously. Such comprehensive attention modeling helps in overcoming the sub-optimal attention learning of global attention, which tends to guide feature learning toward the global salient regions which often suppresses local informative regions around organs.

From the above aspects, we incorporate part-aware multi-granular feature learning and visual attention and make them learn in a collaborative and complementary way.

Since the proposed PMGAN is regularized by the segmentation constraint (as defined in Equation (8)) in the training stage, the segmentation errors brought by off-the-shelf anatomical segmentation techniques may cause the attention learning to deteriorate, and thus lead to sub-optimal thorax disease classification.

## 5. Conclusions

In this paper, we propose a comprehensive thorax disease classification framework, PMGAN, that learns a multi-branch network guided by soft attention and part-aware mask-guided attention. Unlike most existing thorax disease classification methods, which either directly learn global feature representations from whole CXR images or search for global informative regions only, the proposed PMGAN independently captures global and local visual cues from precisely located all-organ and single-organ regions by incorporating soft attention and part-aware mask-guided attention modules, as well as a four-branch network. In addition, a novel multi-task learning strategy is designed that optimizes multiple binary cross-entropy loss on the same disease label concurrently to maximize the learning of complementary global and local branches. Experimental results on the widely-used CXR dataset ChestX-ray14 demonstrate the proposed PMGAN obtains superior thorax disease classification performance against state-of-the-art approaches. Extensive ablation analysis and discussions are also performed to provide more insight into the proposed PMGAN.

## Figures and Tables

**Table 1:** Detailed design and implementation of the baseline network.

Layer #	Stage	Output Size	Layer
1	Conv1	256 × 256	7 × 7, 64, Stride-2
9	Block I	128 × 128	3 × 3 MaxPooling, Stride-2
			$\left[\begin{array}{c} 1\times 1,64\\ 3\times 3,64\\ 1\times 1,256 \end{array}\right]$ × 3
18	Block II	64 × 64	$\left[\begin{array}{c} 1\times 1,256\\ 3\times 3,256\\ 1\times 1,512 \end{array}\right]$ × 6
12	Block III	32 × 32	$\left[\begin{array}{c} 1\times 1,512\\ 3\times 3,512\\ 1\times 1,1024 \end{array}\right]$ × 4
9	Block IV	16 × 16	$\left[\begin{array}{c} 1\times 1,1024\\ 3\times 3,1024\\ 1\times 1,2048 \end{array}\right]$ × 3
1	Prediction	14	Global Average Pooling
			14 Fully Connection

**Table 2:** Comparison with the state of the arts (using global information only) on the dataset ChestXray14: the best performance at each row is shown in bold. ‘*’ denotes that the performance is evaluated by *k*-fold cross-validation method.

Methods	Wang [9]	TieNet [25]	Yao [27]	Gündel [28]	DualCheX Net [24]	AECNN [22]	CheX GCN [26]	CheXNet [23]	Ours	Ours *
**Atelectasis**	70.00	73.20	77.20	78.50	78.40	78.20	78.60	80.90	**84.09**	83.56
**Cardiomegaly**	81.00	84.40	90.40	89.20	88.80	90.10	89.30	**92.50**	92.15	91.90
**Effusion**	75.90	79.30	85.90	83.60	83.10	83.60	83.20	86.40	89.19	**89.34**
**Infiltration**	66.10	66.60	69.50	71.00	70.50	70.90	69.90	**73.50**	72.49	73.06
**Mass**	69.30	72.50	79.20	82.60	83.80	83.80	84.00	86.80	87.24	**88.19**
**Nodul**	66.90	68.50	71.70	75.50	79.60	78.80	80.00	78.00	**83.15**	82.33
**Pneumonia**	65.80	72.00	71.30	73.50	72.70	73.60	73.90	76.80	**78.80**	76.85
**Pneumothorax**	79.90	84.70	84.10	84.70	87.60	86.80	87.60	88.90	91.19	**91.50**
**Consolidation**	70.30	70.10	78.80	74.70	74.60	76.10	75.10	79.00	**81.98**	80.94
**Edema**	80.50	82.90	88.20	83.70	85.20	85.00	85.00	88.80	90.67	**91.25**
**Emphysema**	83.30	86.50	82.90	92.50	94.20	92.20	94.40	93.70	**95.33**	95.06
**Fibrosis**	78.60	79.60	76.70	83.80	83.70	84.00	83.40	80.50	**87.06**	85.26
**PT**	68.40	73.50	76.50	78.50	79.60	78.30	79.50	80.60	81.02	**83.03**
**Hernia**	87.20	87.60	91.40	90.50	91.20	92.40	92.90	91.60	**96.75**	96.24
**Avg**	74.51	77.24	80.27	81.59	82.36	82.41	82.63	84.14	**86.51**	86.32

**Table 3:** Comparison with the state-of-the-art (using both global and local information, or using visual attention) on the dataset ChestXray14: the best performance at each row is shown in bold. ‘*’ denotes that the performance is evaluated by *k*-fold cross-validation method.

Methods	Thorax Net [12]	AG [19]	Ma [17]	SDFN [12]	CAN [20]	$A^{3}$Net [16]	Huang [18]	Ours	Ours *
**Atelectasis**	75.00	75.19	76.27	78.10	77.70	77.90	82.97	**84.09**	83.56
**Cardiomegaly**	87.10	88.42	88.35	88.50	89.40	89.50	91.55	**92.15**	91.90
**Effusion**	81.80	81.22	81.59	83.20	82.90	83.60	88.78	89.19	**89.34**
**Infiltration**	68.20	69.79	67.86	70.00	69.60	71.00	71.15	72.49	**73.06**
**Mass**	79.90	79.56	80.12	81.50	83.80	83.40	86.19	87.24	**88.19**
**Nodul**	71.50	71.72	72.93	76.50	77.10	77.70	80.83	**83.15**	82.33
**Pneumonia**	69.40	69.23	70.97	71.90	72.20	73.70	78.09	**78.80**	76.85
**Pneumothorax**	82.50	85.70	83.77	86.60	86.20	87.80	87.95	91.19	**91.50**
**Consolidation**	74.20	72.30	74.43	74.30	75.00	75.90	81.15	**81.98**	80.94
**Edema**	83.50	83.14	84.14	84.20	84.60	85.50	89.92	90.67	**91.25**
**Emphysema**	84.30	88.60	88.36	92.10	90.80	93.30	93.87	**95.33**	95.06
**Fibrosis**	80.40	78.81	80.07	83.50	82.70	83.80	83.70	**87.06**	85.26
**PT**	74.60	76.19	75.36	79.10	77.90	79.10	79.06	81.02	**83.03**
**Hernia**	90.20	91.38	87.63	91.10	93.40	93.80	94.92	**96.75**	96.24
**Avg**	78.76	79.38	79.41	81.47	81.70	82.57	85.01	**86.51**	86.32

**Table 4:** Ablation study on the dataset ChestXray14: the best performance at each row is shown in bold.

Methods	Baseline	SA	MA
**Atelectasis**	82.29	83.45	**84.09**
**Cardiomegaly**	90.60	91.63	**92.15**
**Effusion**	88.38	88.70	**89.19**
**Infiltration**	70.61	71.55	**72.49**
**Mass**	85.72	86.51	**87.24**
**Nodul**	78.77	80.25	**83.15**
**Pneumonia**	76.71	77.30	**78.80**
**Pneumothorax**	87.49	89.65	**91.19**
**Consolidation**	81.04	81.52	**81.98**
**Edema**	89.29	90.44	**90.67**
**Emphysema**	92.57	93.85	**95.33**
**Fibrosis**	84.92	85.94	**87.06**
**PT**	78.56	80.14	**81.02**
**Hernia**	90.84	94.68	**96.75**
**Avg**	84.18	85.40	**86.51**

**Table 5:** Comparison of mask-guided attention after different block: the best performance at each row is shown in bold.

Methods	Block I	Block II	Block III	Block IV
**Atelectasis**	83.75	83.94	**84.09**	83.79
**Cardiomegaly**	91.12	91.30	**92.15**	91.62
**Effusion**	88.84	88.85	**89.19**	88.87
**Infiltration**	71.75	71.99	**72.49**	71.75
**Mass**	86.74	87.03	**87.24**	87.23
**Nodul**	82.56	82.72	**83.15**	82.50
**Pneumonia**	77.13	78.17	**78.80**	77.51
**Pneumothorax**	90.34	90.89	**91.19**	90.49
**Consolidation**	81.21	81.34	**81.98**	81.55
**Edema**	90.42	90.47	**90.67**	90.26
**Emphysema**	94.83	95.22	**95.33**	94.92
**Fibrosis**	85.83	86.44	**87.06**	86.23
**PT**	80.13	80.54	**81.02**	80.45
**Hernia**	94.61	95.95	**96.75**	93.52
**Avg**	85.66	86.06	**86.51**	85.76

**Table 6:** Comparison of single loss and multiple loss: the best performance at each row is shown in bold.

Methods	Single Loss	Multiple Loss
**Atelectasis**	83.46	**84.09**
**Cardiomegaly**	91.89	**92.15**
**Effusion**	88.95	**89.19**
**Infiltration**	71.96	**72.49**
**Mass**	86.39	**87.24**
**Nodul**	81.59	**83.15**
**Pneumonia**	77.56	**78.80**
**Pneumothorax**	89.84	**91.19**
**Consolidation**	81.57	**81.98**
**Edema**	89.92	**90.67**
**Emphysema**	94.15	**95.33**
**Fibrosis**	85.89	**87.06**
**PT**	80.42	**81.02**
**Hernia**	96.26	**96.75**
**Avg**	85.70	**86.51**

**Table 7:** Comparisons of CPU computational complexity. FLOPs: the number of floating-point operations.

Models	FLOPs	Branch
Baseline	$2.14\times 10^{10}$	1
PMGAN	$4.49\times 10^{10}$	4

**Table 8:** Comparison of different losses for segmentation constraint computing: the best performance at each row is shown in bold.

Methods	BCE	Dice	RMSE
**Atelectasis**	83.78	83.75	**84.09**
**Cardiomegaly**	91.98	92.07	**92.15**
**Effusion**	88.98	88.85	**89.19**
**Infiltration**	72.02	72.28	**72.49**
**Mass**	86.79	86.59	**87.24**
**Nodul**	82.34	82.90	**83.15**
**Pneumonia**	78.18	**78.99**	78.80
**Pneumothorax**	90.59	90.45	**91.19**
**Consolidation**	81.63	81.34	**81.98**
**Edema**	90.28	90.10	**90.67**
**Emphysema**	94.83	95.06	**95.33**
**Fibrosis**	**87.10**	86.72	87.06
**PT**	80.52	81.01	**81.02**
**Hernia**	95.69	96.15	**96.75**
**Avg**	86.05	86.16	**86.51**

## Data Availability

No new data were created or analyzed in this study. Data sharing is not applicable to this article.
